# Cardiac Implantable Electronic Devices Breakthrough: Are We Ready to Face the Future?

**DOI:** 10.3390/jcm11216321

**Published:** 2022-10-26

**Authors:** Gianfranco Mitacchione

**Affiliations:** Cardiology Department, Luigi Sacco University Hospital, Via G.B. Grassi 74, 20157 Milan, Italy; gianfrancomit@hotmail.com

Since its inception cardiac electrical therapy has evolved, with transvenous pacemakers (PMs) and implantable cardiac defibrillators (ICDs) providing significant benefits in terms of improved quality of life and reducing mortality in patients with cardiac conduction disturbances and/or requiring protection against ventricular arrhythmias. Nonetheless, cardiac implantable electronic devices (CIEDs) remain associated with a significant rate of combined short- and long-term system adverse events, such as lead malfunctions, pulse generator pocket complications, and local/systemic infections—with these latter events characterized by high morbidity, protracted antimicrobial therapy, and long-term hospitalization resulting in a substantial financial burden for the healthcare system.

Overwhelmingly, transvenous lead extraction (TLE) has proven to be the most effective solution for CIED-related infective complications and malfunctions, with a high overall efficacy and safety record. There are several tools tailored specifically to remove transvenous devices. 

In order to minimize CIEDs-related adverse events, pacing and high-voltage device manufacturers have recently undergone an impressive technological development, introducing on the market new “*unconventional*” devices, which are characterized by new implant sites and different interactions with intracardiac sites. These new devices are confirming a significant outcome, but in the cases of adverse events the removal can still be challenging. Indeed, it seems that TLE technologies do not keep up with the times in respect to new CIEDs.

## 1. The Advent of Leadless Pacing

Leadless pacemakers (LPMs) have been a major breakthrough in the management of bradyarrhythmia and as an alternative to the standard transvenous PMs. LPM implantation has been steadily increasing over time. To date, the Micra transcatheter pacing system (Micra VR-MC1VR01, Medtronic, Inc., Minneapolis, MN, USA) is the only leadless device available on the market. It was approved by the CE in 2015 and subsequently the FDA approved it in 2016. Recently, the introduction of second generation LPMs (Micra AV-MC1AVR1) has expanded the pacing modes to obtain atrioventricular (AV) synchronous pacing, thus providing an interesting alternative in the scenario of leadless pacing [1].

LPM showed a high safety and efficacy profile when compared to transvenous PMs, with a reduction of 51% in major complications in the early post-procedural period. This is due to the characteristics of the devices’ designs, which avoid complications associated with transvenous leads and surgical pockets [2]. Despite this safety profile, a small percentage of patients still require system revision for device-related adverse events (i.e., premature battery depletion), with the need to optimize electrical features [3].

Recently, serious concerns related to LPMs have been experienced with the Nanostim Leadless Cardiac Pacemaker (St. Jude Medical, St Paul, MN, USA). This device was permanently withdrawn from the market in 2017 for several safety advisories such as battery premature depletion and disfunction of the retrieval catheter. 

The main concern regarding LPMs is that these devices are not designed to be removed; moreover, actual technologies and tools for lead extraction are of limited use for these devices, with scarce experiences reported in the literature [4]. 

On 7 February 2022, Abbott announced the world’s first patient implants of a dual-chamber leadless pacemaker system as part of its AVEIR DR i2i™ pivotal clinical study. The Aveir™ dual-chamber leadless pacemaker (Abbott Cardiovascular Systems Inc., Chicago, IL, USA) is a leadless pacemaker comprising two separate parts, separately screwed in the right atrium and right ventricle that are able to communicate with each other to guarantee AV synchrony. This is a clinical milestone, but there are several concerns about the ability to remove them if required.

## 2. High Voltage Electrical Therapy

Subcutaneous ICD is currently a reasonable solution for patients requiring implantation of a cardioverter defibrillator with no indications of cardiac resynchronization, bradycardia support, or antitachycardia pacing. This device is characterized by the absence of leads in the central venous circulation and inside the cardiac chambers, thus avoiding the risk of vascular obstruction, thrombosis, infection, and cardiac perforation. Therefore, S-ICD is a first line indication in several cases such as pediatric patients, patients with lack of vascular access, or patients at very high risk of infection. Despite the notable safety and efficacy profile, cases of device-related complications (unappropriated shocks and/or local infections) have been reported [5]. First experiences on S-ICD lead extraction require specific tools, specifically when fibrotic adhesions have developed around the parasternal coil [6].

To date, the only S-ICD available on the market is the Emblem^TM^ MRI S-ICD system (Boston Scientific, Marlborough, MA, USA). The system was approved by the FDA in 2012, although some series were recalled in 2021 due to premature battery depletion. The lead, with an 8 cm shock coil, is vertically positioned in the subcutaneous tissue of the chest, parallel to and 1–2 cm from the left sternal midline followed by a horizontal segment until it reaches the left anterior axillary line. First experiences on S-ICD lead extraction are encouraging [5]; however, they require specific tools, especially when fibrotic adhesions develop around the parasternal coil. 

A new S-ICD system has been developed (EV ICD™ System. Medtronic, Inc., Minneapolis, MN, USA). The new system is characterized by a single lead implanted under the sternum that can pace patients out of ventricular tachycardia (but not bradycardia). This specifical feature could also play a negative role in case of device removal, particularly after a consistent time. 

Finally, leadless cardiac pacing and subcutaneous defibrillator technologies are about to be merged. In fact, Boston Scientific recently presented (American Heart Association Scientific Sessions 2021) preclinical data on the EMPOWER MPS device, a leadless pacemaker that is able to communicate with the Emblem^TM^ S-ICD. This promises not only to deliver antitachycardia pacing therapy, but also to pace in VVIR mode. 

## 3. Conclusions

Several technological progresses have been made in order to minimize CIEDs-related adverse events. These translate into the development of miniaturized CIED, avoiding as much as possible any interaction with intracardiac tissues. Unfortunately, adverse events from these devices still remain. In this field, further efforts are needed to improve the safety of the devices and develop new techniques that could overcome these unresolved issues, particularly in the field of “new” CIEDs extraction. 

## References

[1] Mitacchione G., Schiavone M., Gasperetti A., Viecca M., Curnis A., Forleo G.B. (2021). Atrioventricular synchronous leadless pacemaker: State of art and broadened indications. Rev. Cardiovasc. Med..

[2] Piccini J.P., Stromberg K., Jackson K.P., Laager V., Duray G.Z., El-Chami M., Ellis C.R., Hummel J., Jones D.R., Kowal R.C. (2017). Long-term outcomes in leadless Micra transcatheter pacemakers with elevated thresholds at implantation: Results from the Micra Transcatheter Pacing System Global Clinical Trial. Heart Rhythm.

[3] Mitacchione G., Arabia G., Schiavone M., Cerini M., Gasperetti A., Salghetti F., Bontempi L., Viecca M., Curnis A., Forleo G.B. (2022). Intraoperative sensing increase predicts long-term pacing threshold in leadless pacemakers. J. Interv. Card. Electrophysiol..

[4] Curnis A., Cerini M., Mariggiò D., Mitacchione G., Giacopelli D., Inama L., Bontempi L. (2019). First-in-human retrieval of chronically implanted Micra transcatheter pacing system. PACE Pacing Clin. Electrophysiol..

[5] Mitacchione G., Schiavone M., Gasperetti A., Viecca M., Curnis A., Forleo G.B. (2020). Neglected lead tip erosion: An unusual case of S-ICD inappropriate shock. J. Cardiovasc. Electrophysiol..

[6] Behar N., Galand V., Martins R.P., Jacon P., Badenco N., Blangy H., Alonso C., Guy-Moyat B., El Bouazzaoui R., Lebon A. (2020). Subcutaneous Implantable Cardioverter-Defibrillator Lead Extraction: First Multicenter French Experience. JACC Clin. Electrophysiol..

